# Sexual Dimorphism in the Polarization of Cardiac ILCs through Elabela

**DOI:** 10.3390/cimb45010017

**Published:** 2022-12-30

**Authors:** Évila Lopes Salles, Sahar Emami Naeini, Bidhan Bhandari, Hesam Khodadadi, Edie Threlkeld, Sholeh Rezaee, William Meeks, Avery Meeks, Aderemi Awe, Ahmed El-Marakby, Jack C. Yu, Lei P. Wang, Babak Baban

**Affiliations:** 1 Department of Oral Biology and Diagnostic Sciences, Dental College of Georgia, Augusta University, Augusta, GA 30912, USA; 2 Medical College of Georgia, Augusta University, Augusta, GA 30912, USA; 3 Department of Plastic Surgery, Medical College of Georgia, Augusta University, Augusta, GA 30912, USA; 4 Medicinal Cannabis of Georgia LLC, Augusta, GA 30912, USA

**Keywords:** Elabela, CD28, ILCs, innate immunity, sexual-dimorphism, heart immunity, cardiac ILCs

## Abstract

Elabela is a component of the apelinergic system and may exert a cardioprotective role by regulating the innate immune responses. Innate lymphoid cells (ILCs) have a significant role in initiating and progressing immune-inflammatory responses. While ILCs have been intensively investigated during the last decade, little is known about their relationship with the apelinergic system and their cardiac diversity in a gender-based paradigm. In this study, we investigated the polarization of cardiac ILCs by Elabela in males versus females in a mouse model. Using flow cytometry and immunohistochemistry analyses, we showed a potential interplay between Elabela and cardiac ILCs and whether such interactions depend on sexual dimorphism. Our findings showed, for the first time, that Elabela is expressed by cardiac ILCs, and its expression is higher in females’ ILC class 3 (ILC3s) compared to males. Females had higher frequencies of ILC1s, and Elabela was able to suppress T-cell activation and the expression of co-stimulatory CD28 in a mixed lymphocyte reaction assay (MLR). In conclusion, our results suggest, for the first time, a protective role for Elabela through its interplay with ILCs and that it can be used as an immunotherapeutic target in the treatment of cardiovascular disorders in a gender-based fashion.

## 1. Introduction

Elabela is a hormonal peptide, one of the two endogenous ligands for the G-coupled receptor called APJ (Apelin receptor, APLNR) [1]. The gene from which Elabela is translated is called the Apela or Ela gene and is located on chromosome 4. During its process of synthesis, Elabela is founded in the Golgi apparatus as a full length peptide with 54 amino acids. However, inside the Golgi this peptide is cleaved into its mature form with 32 amino acids and then forwarded for secretion [2,3]. The activation of the APJ receptor by Elabela is related to a wide range of physiological effects. The ELA-APJ receptor signaling has been reported to lead to heart development, anti-fibrotic effects, angiogenesis, anti-inflammatory response, and cardiovascular protection [1,2]. Importantly, Elabela is widely expressed in the cardiovascular system of fetuses and adults. In adults, the low level of Elabela in the plasma may be involved in the pathogenesis of hypertension and vascular damage [4]. According to the literature, Elabela could alleviate adverse vascular remodeling and prevent myocardial fibrosis and dysfunction through activation of PI3K, Akt, and ERK1/2, which would promote anti-inflammatory, anti-oxidative, and anti-proliferative effects [5,6,7], although the mechanisms are still not fully understood.

Innate lymphoid cells (ILCs) are considered the architects of the immune system. They are tissue-resident innate immune cells with big diversity and functional plasticity and are still being investigated. There are three major groups of ILCs that are distinguished based on three main aspects: (1) the cytokines they produce, (2) the transcription factors involved in their differentiation, and (3) their phenotypic markers. T-bet+ ILC1s produce interferon-γ (IFN-γ); GATA3+ ILC2s secrete interleukin-5 (IL-5), IL-9, IL-13, and amphiregulin; and Rorγt+ ILC3s produce IL-22 and IL-17 [8,9]. The ILCs play a key role in several physio-pathological processes, from organogenesis during development [8] to tissue repair and protection against pathogens and maintenance of homeostasis [10,11].

The unique aspects of ILCs enable these cells to respond not only to pathogens but also to sense signals from neuropeptides, hormones, and metabolites [12]. Despite extensive investigations of ILCs, there is yet long way to be paved before the role of ILCs in cardiovascular tissues could be fully elucidated. ILCs play an important role in the pathogenesis of atherosclerosis, myocardial infarction, and peripheral artery diseases, among all other cardiac disorders [13,14]. Pre-clinical studies in mouse model have indicated that ILC2s is the major source of IL-5 and IL-3, which are extremely important to sustain atheroprotective, immunity-limiting vascular inflammation and atherosclerotic lesion development [15].

Here in this study, we investigated for the first time, a potential relationship between Elabela and cardiac ILCs in a sex-dependent fashion. We rationalized our studies based on: (1) the immune-regulatory potential of peptides [16], (2) lower incidence of premenopausal women in developing cardiovascular diseases compared to their male counterparts [17], and (3) existing variations in immune response due to genetic (linked to X chromosome) and hormonal differences. The outcomes of current studies could help better understand the complex roles of the immune system and its interplay with physiologic mediators as well as more effective treatment of cardiovascular diseases in a gender-based manner.

## 2. Results

### 2.1. Cardiac ILCs Are Polarized in a Sex-Dependent Fashion

**Figure 1:**
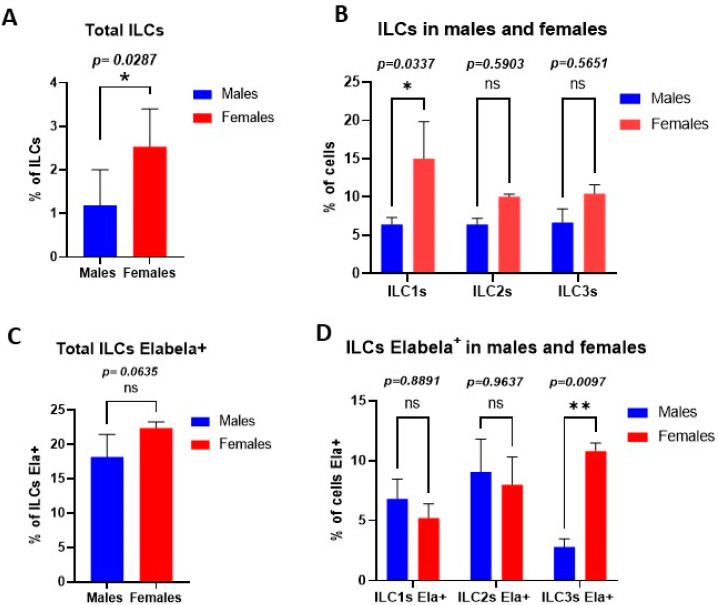
ILCs polarization in a sex-dependent fashion in cardiac tissues. (**A**) Percentage of ILCs among the total cells analyzed by flow cytometry. (**B**) Percentage of each class of ILCs in total ILCs, ILC1s were significantly higher in the female cardiac tissues than male counterparts. (**C**) Percentage of each class of ILCs that express Elabela; female cardiac ILC3s showed significantly higher levels of Elabela compared with male counterparts. Statistical tests: t-test followed by Mann–Whitney (**A**,**C**) and two-way ANOVA followed by Šidák multiple comparisons test (**B**,**D**). * *p ≤* 0.05; ** *p ≤* 0.01.

The flow cytometry results showed that there is a difference in the total percentage of ILCs between males (1.8% of cells) and females (2.53% of the cells) (Figure 1A). Furthermore, the percentage of the classes of ILCs was different in males versus females. Females’ hearts contained more ILC1s (15% of cells) compared to males (6.4% of cells). No other significant differences were observed between other classes of cardiac ILCs in males versus females (Figure 1B).

### 2.2. Expression of Elabela by Cardiac ILCs in a Sex-Dependent Manner

Our findings showed that there is no difference in the total percentage of Elabela expressing ILCs between males (18.2% of cells) and females (22.4% of cells) (Figure 1C). While all classes of cardiac ILCs were expressing Elabela, the highest level of Elabela expression was seen in female cardiac ILC3s (10.8% of ILCs Elabela+), which was significantly different from male their counterparts (2.8% of cells) (Figure 1D).

### 2.3. Elabela Inhibits T-Cell Activation

**Figure 2:**
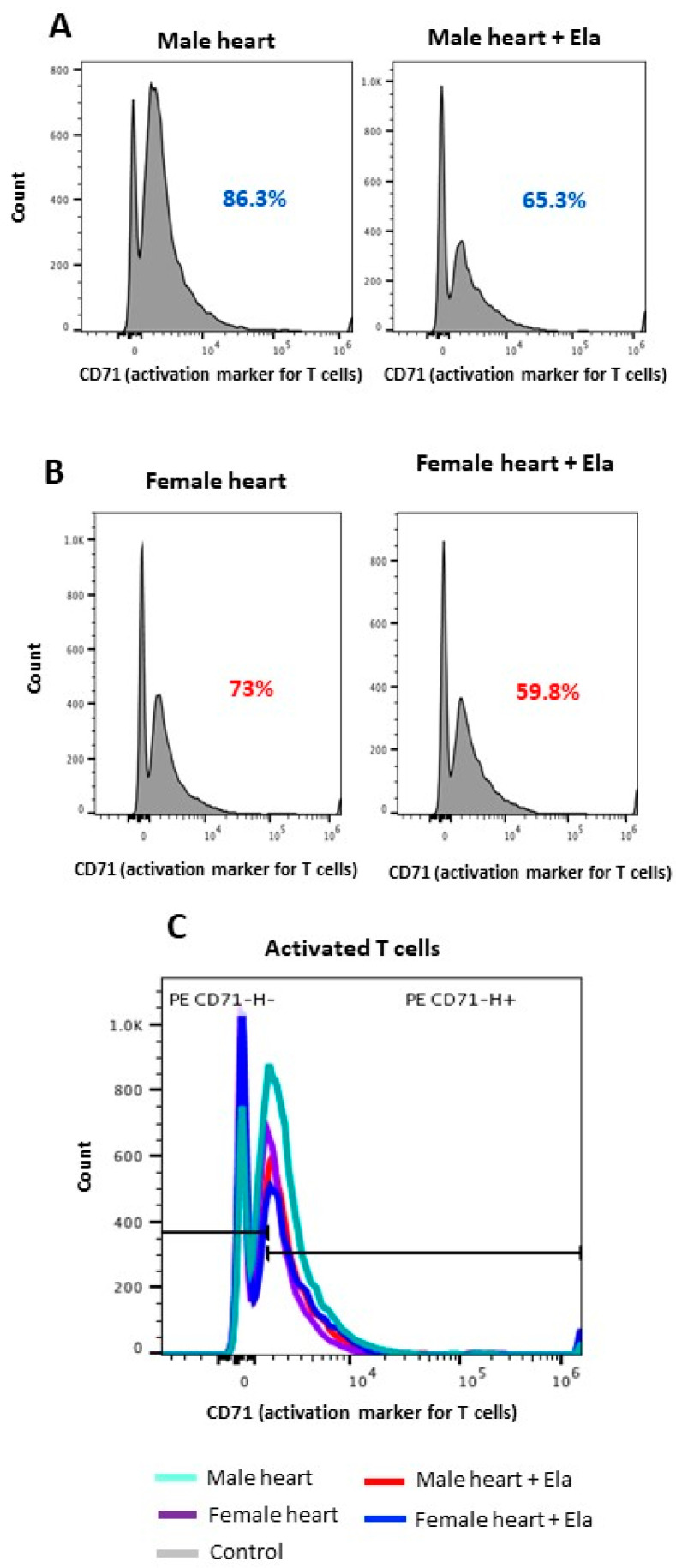
Elabela could suppress T-cell activation. T-cell activation was measured in an MLR assay using male (**A**) and female (**B**) cardiac cells as stimulators versus splenic T cells as responders. Using flow cytometry, CD71-expressing T cells were identified and quantified as T-cell activation markers. (**C**) Histogram panel depicting the comparative analysis of activated T cells in males versus females.

The mixed lymphocyte reaction assay (MLR) showed that Elabela could effectively suppress T-cell activation. Such an effect was higher when cardiac cells from males were used as stimulators (20% suppression) compared to female cells (14% suppression) (Figure 2A,B), suggesting a sex-dependent effect of Elabela on T-cell activation. Figure 2C shows the histogram from flow cytometry analysis for the expression of CD71, a marker for T-cell activation.

### 2.4. Differential Expression of CD28 in Male and Females’ Heart

**Figure 3:**
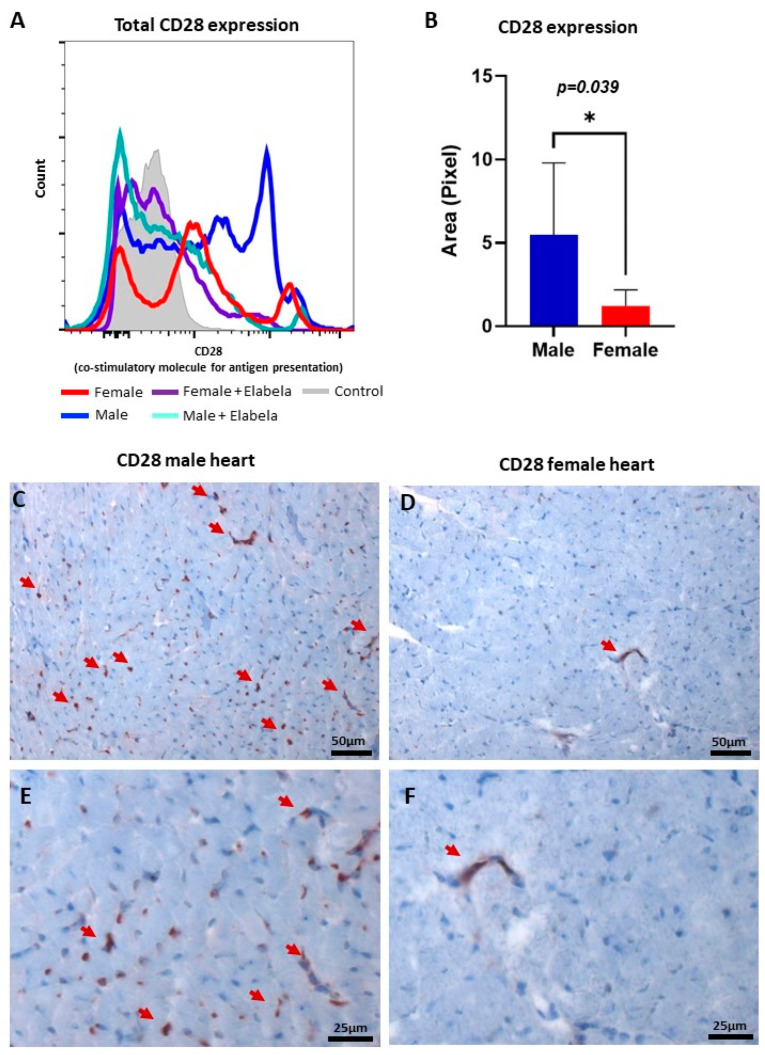
Regulation of CD28 co-stimulatory protein by Elabela in a sex-dependent fashion. (**A**) Histogram panel of flow cytometry analysis demonstrated that Elabela could reduce the expression of CD28 in T cells, modulating their activation. (**B**) Area in pixel for CD28 expression in male versus female normal heart tissues, quantifying the expression level in cardiac tissues of both sexes using Immunohistochemistry. Microscopic images showing the presence of CD28 (brown color) in male (**C**) and female (**D**) hearts, magnification 200×. (**E**,**F**) Shown in higher magnification (400×), the expression of CD28 in male and female hearts, respectively. The arrows point to cells positive for CD28. * *p ≤* 0.05.

Since CD28 is a primary costimulatory molecule for T-cell activation, a flow cytometry analysis of the CD28 expression was performed (Figure 3). The MLR results showed that the expression of CD28 was 5-fold higher in males than females (Figure 3A). When Elabela was added to the medium, the expression of CD28 decreased significantly in males. The histology analysis showed that in normal hearts the expression of CD28 in the myocardium was significantly higher in males (Figure 3B) than females (Figure 3C). This result might explain why the activation of T cells was different between males and females. Additionally, this result opens a new potential pathway and role for Elabela in the modulation of the immune system.

## 3. Discussion

Males and females differ in their innate and adaptive immune responses. These differences have important implications for the development and trajectory of diseases [18]. Consistent with such notions, our findings here in this study showed a variance in cardiac ILCs between male versus female mice. Our observations revealed that female mice had more ILC1s compared to male mice. ILC1s are a heterogeneous group that can act through several mechanisms to regulate the inflammation in the tissue [10]. Given the crucial role of ILCs in the initiation and progression of the immune system, the higher frequency of cardiac ILC1s in female mice could suggest a reasonable justification for females to be more protected against infections compared to male counterparts [6,19]. Interestingly, our data were not in agreement with a prior study reporting that ILC2s were a dominant type of ILCs in both male and female cardiac tissues [20]. It is well established that genetic diversity and environmental conditions may greatly influence the immune profile and inflammatory responses in laboratory animals [21]. Therefore, such discrepancy in ILCs profile might well be due to the type and genetic background of the mice used in the studies. While we used ICR mice in our study, the previous report had used C57BL/6 mice.

The hallmark of our study was to show, for the first time, that cardiac ILCs express Elabela and that such expression was gender-dependent. Females demonstrated a higher frequency of ILC3s expressing Elabela compared to their male counterparts. ILC3s are known for their plasticity and potential dichotomic functions, contributing to maintaining and re-establishing homeostasis in several inflammatory diseases [22,23,24]. Therefore, it is plausible to propose that Elabela can influence ILC3s signaling in response to a variety of stimuli, determining the direction of the ILC3s response, which potentially affects the immune balance within the tissue microenvironment. It is worth mentioning that Elabela shows anti-inflammatory and anti-fibrotic activity in several organs, including the heart [25,26]. Further, the higher expression of Elabela by ILC3s may affect the whole profile of cardiac ILCs by altering the plasticity and polarization of ILC3s, re-formatting the dynamic of tissue microcosm and cardiac homeostasis. Importantly, several studies have reported the central role of ILC3s in establishing homeostasis and immune tolerance within a variety of tissues through effective interaction with adaptive immunity [27,28]. On this point, it is proposed that ILC3s express program cell death ligand 1 (PDL1), engaging with program death 1 (PD1) on effector T cells, causing cell death and suppression of T-cell activation [29]. Our findings showed for the first time that Elabela could reduce the expression of CD28, affecting co-stimulatory signaling and T-cell activation negatively. The low expression of CD28 would impair the process of antigen presentation between CD28 T cells and CD80/CD86 antigen-presenting cells, which would inhibit T-cells activation and production of pro-inflammatory cytokines and chemokines [30]. Such regulatory impact by ILC3s expressing Elabela may be an immune therapeutic target in the treatment of inflammatory responses within cardiac as well as other tissues.

According to the literature, Elabela would be able to control inflammation through the activation of the APJ receptor. The activation of APJR by Elabela would downregulate ACE and promotes ACE2 activity. ACE2 has an anti-inflammatory role and decreases the expression of interleukin-6 (IL-6), interleukin-1β, and tumor necrosis factor α (TNFα), which are known for their pro-inflammatory activity [31]. Additionally, Zhang and colleagues, explain that Elabela treatment would be able to reduce renal inflammation induced by diabetes through the APJ receptor and activation of the PI3k/Akt/mTOR pathway. The activation of the PI3K/Akt/mTOR signaling pathway decreased the expression levels of pro-inflammatory monocyte chemoattractant protein-1 (MCP-1), intercellular adhesion molecule 1 (ICAM-1), and TNF-α, decreasing inflammation [5]. In this study, we propose that Elabela regulates inflammation also through the modulation of T-cell activation.

Additionally, our analysis demonstrated higher expression of CD28 in male cardiac tissues than in that of females. Given the capacities of both Elabela [26] and CD28 [32] in activation of the PI3K/Akt pathway, further research is warranted to investigate the potential therapeutic role of Elabela expressing ILCs, manipulating such dichotomic paradigm of activation and inhibition during inflammatory responses in a gender-based fashion. In fact, sexual dimorphism of Elabela expression and its interplay with ILCs may help better understand and explain the low incidence of heart diseases in females compared to their male counterparts [33]. The investigation of the mechanisms we proposed will open new targets for more accurate prevention, diagnosis, and treatment of heart diseases according to the patient’s biological sex.

It is important to highlight the limitation of our work. The low frequency of ILCs and the complexity of ILCs assessment through histology and immunohistochemical analysis are examples of major limitations in our studies. Further, the lack of sufficient data on APJ expression and the interactions with Elabela in tissues and in relation to ILCs could be added to the limitations list, warranting further research. Although the consistency of ILCs’ roles [34] and Elabela’s functions [35] in mice and humans have been reported, we need to consider that some mechanisms may vary and so need to be better investigated.

In conclusion, our current data is the first report to show the expression of Elabela by ILCs in cardiac tissues. Given the central role of ILCs in the initiation, regulation, and resolution of inflammatory diseases, such expression of Elabela may suggest a mechanistic link for ILCs with translational values in the treatment of cardiac injuries. More importantly, considering the crucial role of T-cell activation during the remodeling process of cardiac tissues [36], Elabela expression by ILCs could be targeted as an immunotherapeutic modality in the treatment of cardiac injuries. Further, as suggested by our novel findings here in this study, sex dimorphism may affect such potential in a profound way, warranting further research.

## 4. Materials and Methods

### 4.1. Animals

The hearts of adult (12 weeks) male and female ICR mice (*n* = 5 mice/group) were harvested and subjected to flow cytometry, immunofluorescence, and histological analysis.

### 4.2. Flow Cytometry

For analytical flow cytometry, single-cell suspension was prepared from the heart. In brief, tissue samples were sieved through a 100 μM cell strainer (BD Biosciences, San Diego, CA, USA), followed by centrifugation (252× *g*, 10 min) to prepare single-cell suspensions. The samples were incubated with CD45, CD3, CD24, CD11b (lineage), and CD127. Next, cells were fixed and permeabilized using fix/perm concentrate (eBioScience) before incubation with antibodies for intracellular staining with T-bet (ILC1s); Gata3 (ILC2s); RoRγt (ILC3s); and Elabela (Phoenix Pharmaceutical, Inc., cat. H-007-19, Burlingame, CA, USA). Cells were then run through a 4-Laser LSR II flow cytometer. Cells were gated based on forward and side scatter properties and on marker combinations to select cells of interest. All acquired flow cytometry data were analyzed using FlowJo V10. Graphs and summary statistics were also used to assess the results.

### 4.3. Mixed Lymphocyte Reactions (MLR)

To assess the regulatory function of Elabela, we used an MLR assay as described previously with minor modifications [37] in an in-vitro and sex-dependent setting. MLR is a valuable method to evaluate immunomodulatory drugs, anti-inflammatory agents, and implantable materials. Cardiac cells from female and male ICR donors were used as stimulator (Antigen Presenting Cells) and incubated with T cells (responders) from an HLA mismatch C57BL/6 mouse spleen in a ratio of 5:1 stimulator to responders. Briefly, responder T lymphocytes were initially enriched using Magnetic Assorted Cell System (MACS) plated at 1 × 10^4^ cells per well. Cardiac cell suspension was prepared by sieving the cardiac tissues through micro cell strainers as described previously [37]. These cardiac cells were then used as stimulators following plating at 5 × 10^4^cells per well. Combinations of responders (naive T lymphocytes) and stimulators (cardiac cells) were prepared in triplicate wells with Elabela or vehicle. Cells were cultured in 200 μl per well of RPMI 1640 medium supplemented with FBS, penicillin, streptomycin, L-glutamine, and 2-ME. After 72–96 h of incubation at 37 °C in a humidified 5% CO_2_ incubator, cells were harvested into flow cytometry tubes. Following a PBS wash, samples were incubated at 4 °C for 20 min in the dark with anti-CD71-PE-conjugated and CD3 antibodies to label activated and dividing T cells. Samples were then washed with PBS, and T-cell proliferation was quantified in triplicate by flow cytometry and analyzed for T-cell activation.

### 4.4. Immunohistochemistry

Multiple 5 μm midcoronal paraffin-embedded sections of heart were deparaffinized in xylene and rehydrated in graded alcohol solutions. Endogenous peroxidase was quenched with 3% H_2_O_2_ in PBS 1x. The sections were then incubated with proteinase K (Dako, Glostrup, Denmark, cat. S3020). The sections were then incubated with anti-murine CD28. Preparations were counterstained with hematoxylin and mounted in Faramount and imaged by bright-field microscopy. The expression of CD28 was quantified by using particle count and color intensity measurements in the ImageJ software version 1.53.

### 4.5. Statistics

In order to determine the statistical differences among the means of all experimental groups, data were analyzed using Graphpad Prism 9. A two-way analysis of variance (ANOVA) followed by Šidák multiple comparisons test was performed to establish significance (*p* < 0.05) among all groups. The particle count and color intensity measurements were analyzed using an unpaired *t*-test.

## Data Availability

Not applicable.
